# P-860. Managing Carbapenem-Resistant *Enterobacterales* (CRE) Bacteremia: Real World Data and Insights from a Quaternary Care Hospital in the United Arab of Emirates

**DOI:** 10.1093/ofid/ofae631.1052

**Published:** 2025-01-29

**Authors:** Rama Nasef, Rania M El Lababidi, Claude Afif

**Affiliations:** Cleveland Clinic Abu Dhabi, Abu Dhabi, Abu Dhabi, United Arab Emirates; Cleveland Clinic Abu Dhabi, Abu Dhabi, Abu Dhabi, United Arab Emirates; Cleveland Clinic Abu Dhabi, Abu Dhabi, Abu Dhabi, United Arab Emirates

## Abstract

**Background:**

Infections caused by carbapenem-resistant *Enterobacterales* (CRE) represent significant clinical challenges due to their poor outcomes and high mortality. Real-world data on the treatment of carbapenem-resistant *Enterobacterales* (CRE) bacteremia in the United Arab of Emirates (UAE) is currently scarce. Our aim was to outline the various treatment approaches for CRE and their respective outcomes

**Figure:**
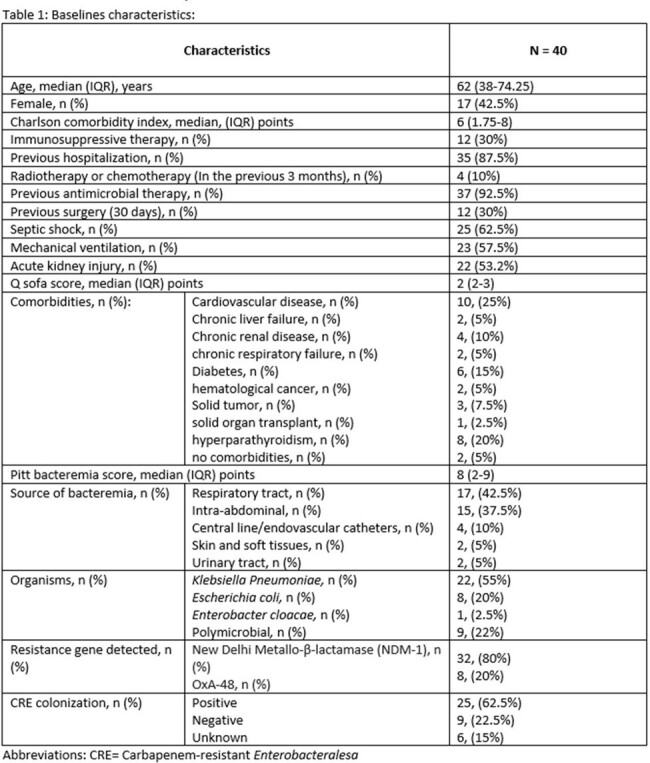

**Methods:**

A retrospective observational study was conducted at our quaternary hospital between July 2021 and March 2024. We included patients with bacteremia caused by CRE infections. Antimicrobial resistance genes were detected using blood culture identification panel (Biofire FilmArray). Our primary outcome was all cause mortality and secondary outcomes were clinical success and 30-days-admission rates. Clinical success was defined as microbiological cure in addition to resolution of symptoms. Data was analyzed through descriptive statistical methods.

**Figure:**
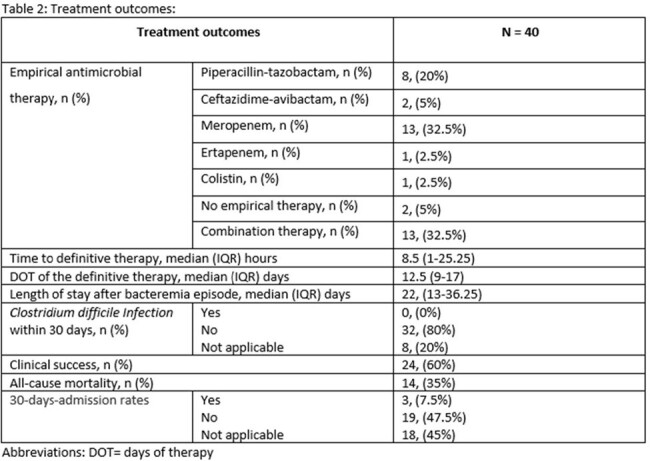

**Results:**

A total sample of 40 patients met the inclusion criteria, 23 males, median age of 62 (38-74.2) years and a median Charlson Comorbidity Index of 6 (1.75-8). The New Delhi Metallo-β-lactamase (NDM-1) resistance gene accounted for (32/40, 80%) of patients, of which, (19/32, 59.3%) were treated with the combination of Ceftazidime-avibactam-Aztreonam. All cause mortality was observed in (14/40, 35%) patients. The mortality observed in patients with NDM resistance gene was (12/32, 37.5%). Over all clinical success and 30-days-admission rates were (24/40, 60%) and (3/40, 7.5%) respectively.

**Figure:**
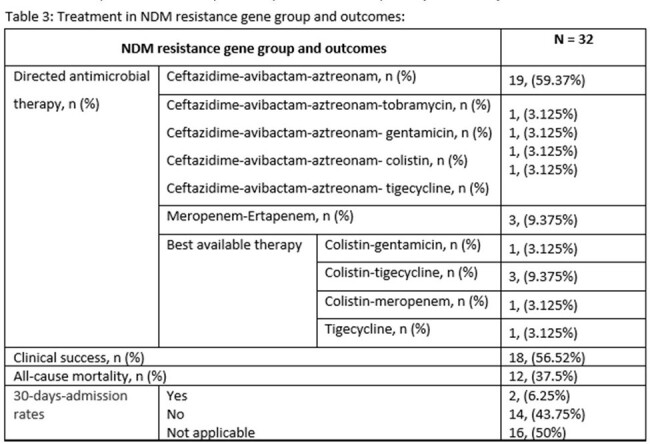

**Conclusion:**

In our cohort of patients with CRE bacteremia, we observed high rates of NDM resistance gene. The overall mortality rate was high at 35% which is similar to what is reported in the literature. Data is urgently needed on the management of CREs with NDM resistance gene to improve patient outcomes.

**Figure:**
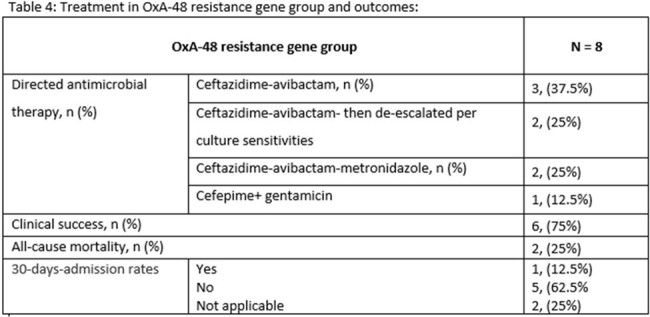

**Disclosures:**

**All Authors**: No reported disclosures

